# In Vitro Shoot Multiplication and Rooting of ‘Kashan’ and ‘Hervy Azerbaijan’ Damask Rose (*Rosa damascena* Mill.) Genotypes for Cosmetic and Ornamental Applications

**DOI:** 10.3390/plants13101364

**Published:** 2024-05-14

**Authors:** Behzad Kaviani, Bahareh Deltalab, Dariusz Kulus, Amir Ali Khoddamzadeh, Cesar Augusto Roque-Borda

**Affiliations:** 1 Department of Horticultural Science, Rasht Branch, Islamic Azad University, Rasht 41335-3516, Iran; 2 Department of Agroecology, Razi University, Kermanshah 671441-4971, Iran; bddaltalab@gmail.com; 3 Laboratory of Horticulture, Faculty of Agriculture and Biotechnology, Bydgoszcz University of Science and Technology, Bernardyńska 6, 85-029 Bydgoszcz, Poland; 4 Department of Earth and Environment, Institute of Environment, Florida International University, Miami, FL 33199, USA; akhoddam@fiu.edu; 5 Vicerrectorado de Investigación, Universidad Católica de Santa María, Arequipa 04000, Peru; cesar.roque@unesp.br

**Keywords:** acclimatization, Damask rose, ornamental plants, plant growth regulators, tissue culture

## Abstract

The damask rose (*Rosa damascena* Mill.) is an ornamental–medicinal plant from the Rosaceae family, and its aromatic compounds and essential oils are applied globally in the food, cosmetic, and pharmaceutical industries. Due to its economic value, this research aimed to establish a protocol for an efficient, rapid, and cost-effective method for in vitro shoot multiplication and rooting of the *R. damascena* ‘Kashan’ and ‘Hervy Azerbaijan’ genotypes. Nodal segments (as primary explants) were cultured on the Murashige and Skoog (MS) medium with combinations of various plant growth regulators (PGRs) such as gibberellic acid (GA_3_), 6-benzylaminopurine (BAP), and indole-3-butyric acid (IBA), as well as a PGR-like substance, phloroglucinol (PG), vitamins such as ascorbic acid (AA), and activated carbon in the form of active charcoal (AC). For the establishment stage, 0.1 mg·L^−1^ PG, 0.2 mg·L^−1^ GA_3_, and 1 mg·L^−1^ BAP were added to the media. Secondary explants (nodal segments containing axillary buds produced from primary explants) were obtained after 30 days of in vitro culture and transferred to the proliferation media supplemented with different concentrations of BAP (0, 0.5, 1, 1.5, 2, and 2.5 mg·L^−1^) and GA_3_ (0, 0.1, 0.2, 0.4, 0.8, and 1 mg·L^−1^) together with 0.1 mg·L^−1^ PG and 20 mg·L^−1^ of AA. The rooting media were augmented with different concentrations of BAP and GA_3_ with 0.1 mg·L^−1^ of IBA, PG and 20 mg·L^−1^ of AA and AC. The results showed that the highest regeneration coefficient (4.29 and 4.28) and the largest number of leaves (23.33–24.33) were obtained in the explants grown on the medium supplemented with 2 mg·L^−1^ BAP and 0.4 mg·L^−1^ GA_3_ for the ‘Kashan’ and ‘Hervy Azerbaijan’ genotypes, respectively. Likewise, this PGR combination provided the shortest time until bud break (approximately 6.5 days) and root emergence (approximately 10 days) in both genotypes. The highest number of shoots (4.78 per explant) and roots (3.96) was achieved in this medium in the ‘Kashan’ rose. Stem and root lengths, as well as stem and root fresh and dry weights, were also analyzed. In most measured traits, the lowest values were found in the PGRs-free control medium. Rooted plantlets were transferred to pots filled with perlite and peat moss in a 2:1 proportion and were acclimatized to ambient greenhouse conditions with a mean 90.12% survival rate. This research contributes significantly to our understanding of Damask rose propagation and has practical implications for the cosmetic and ornamental plant industries. By offering insights into the manipulation of regeneration processes, our study opens up new possibilities for the effective production of high-quality plant material.

## 1. Introduction

The rose is among the most important ornamental species in the world and is sold as a cut, potted, garden, and landscape plant. Roses are also important in the food, pharmaceutics, and medicine industries, and are available as a broad range of cultivars and genotypes in international markets [1]. Moreover, rose petals are a natural source of fragrance and essential oils, which are valuable products in the perfume industry [2,3]. The genus *Rosa* covers over 200 species but only a few are being used for essential oil production. Among them, the Damask rose (*Rosa damascena* Mill.), a hybrid species derived from *Rosa gallica* L. and *Rosa moschata* Herrm, is mainly planted for industrial oil production [4]. Roses are mostly propagated vegetatively; however, the low rooting ability of stem cuttings is the main limiting factor in conventional propagation [5]. Furthermore, plants produced in vivo have poor and uneven oil contents and yield [4]. Consequently, the in vitro propagation of roses has played a critical role in the fast multiplication of species and cultivars with desirable characteristics and in the production of healthy plants [6].

In vitro propagation is acknowledged as an important and effective technique for the large-scale propagation of horticultural crops, as well as for overcoming problems caused by heterogeneous seed production. The success of in vitro propagation techniques depends on a number of factors, i.e., the type and concentration of plant growth regulators (PGRs), the plant’s genotype, the medium type, and explant parameters, which should be carefully optimized [7,8]. Micropropagation techniques have been utilized for several rose species [1,8,9,10] and their hybrids [11]. The successful use of conventional PGRs (auxins and cytokinins) for shoot multiplication and root production in Rosaceae members has been reported [7,10,11]. Cytokinins (6-benzylaminopurine—BAP, or kinetin—KIN) play a pivotal role in micropropagation by promoting cell division and shoot formation, thereby stimulating the development of multiple shoots from explants. Auxins, such as indole-3-acetic acid (IAA), indole-3-butyric acid (IBA), and naphthaleneacetic acid (NAA), on the other hand, stimulate the initiation and development of adventitious roots [9]. Successful in vitro propagation of Damask rose was obtained through the use of nodal explants and a modified Murashige and Skoog (MS) medium with 0.25 mg·L^−1^ IBA and/or 0.5–4 mg·L^−1^ BAP [5,12]. Bosh et al. [13] developed a temporary immersion system for Damask roses based on the use of a modified MS medium with 6 mg·L^−1^ BAP. Nonetheless, studies on the use of other types of PGRs and their interactions in the micropropagation of this species are missing. Performing such research is important since, according to Rezanejad et al. [14], old rose species (such as *R. damascena*) are more difficult to propagate than modern potted cultivars. Another drawback of the research related to micropropagation of the Damask rose is the lack of information on the name of the cultivar studied [5,8,13,14,15]. This requires clarification, especially for commercial purposes, as the effect of genotype on the micropropagation efficiency is significant [10]. 

Phloroglucinol (PG), or 1,3,5-trihydroxybenzene, demonstrates both auxin- and cytokinin-like activity, similar to thidiazuronu (TDZ), and thus has considerable potential in a wide range of plant tissue culture studies [16]. This PGR-like substance is a phenolic compound, a degradation product of phloridzin, and is known to promote the proliferation, growth, and in vitro development of numerous plant species [15]. Our previous study on the ‘Isfahan’ rose genotype focused on indicating the optimal concentration of PG (0, 0.1, 0.2, and 0.3 mg⋅L^−1^) for enhancing shoot multiplication and rooting in combination with BAP (0, 1, 2, and 3 mg⋅L^−1^) and IBA (0, 0.1, 0.2, and 0.3 mg⋅L^−1^) [17]. Based on those findings, here we used a constant concentration of PG and IBA (0.1 mg·L^−1^) in combination with various concentrations of gibberellins and BAP for both shoot and root regeneration in an extended number of genotypes.

Gibberellins (GAs), pentacyclic diterpene acid-derivatives, are naturally occurring plant hormones that are used as PGRs to regulate various biological processes and stimulate both cell division and elongation that affect stem and leaf growth [18,19]. GAs play a fundamental role in many cellular events, such as overcoming dormancy in seeds and buds, and stimulating floral induction, i.e., they are the germination-promoting group of PGRs [20]. Plants maintain their cellular homeostasis by regulating the expression of GAs biosynthesis genes or GAs catabolic genes [21,22]. GA-mediated signaling exhibits a crosstalk with other PGRs such as auxins [23]. Misra and Chakrabarty [24] reported that cytokinins alone were able to induce shoot buds in *Rosa clinophylla* Thory, but the production of these buds was enhanced when cytokinins were used together with GA_3_. Hence, it is interesting to use this PGR in tissue culture systems for the Damask rose.

The ‘Kashan’ genotype is one of the most important genotypes for extracting essential oil and rose water from roses in Iran due to its high performance and excellent aroma. This valuable genotype has a 15% higher essential oil yield and maintains more aromatic compounds in the essential oil compared to other genotypes, and there are 4.6% less undesirable waxy compounds in its essential oil than other genotypes [25]. Plants obtained from the in vitro cultivation of *Rosa damascena* (commercial cultivars and genotypes) have a higher yield of about 10 to 15 tons per hectare compared to plants obtained using the traditional methods (sucker and cuttings) with a yield of 3 to 5 tons per hectare [26]. The micropropagated stocks are of high quality and free from any contamination. At present, the ‘Hervy Azerbaijan’ rose genotype is propagated in the Azerbaijan province, Iran, traditionally through cuttings. The comparison of the tissue culture production of the ‘Kashan’ and ‘Hervy Azerbaijan’ genotypes is justified for the needs of rose production in East Azerbaijan region, Iran. Therefore, the present study aimed to assess the effect of different combinations of BAP, GA_3_, PG, and IBA in the micropropagation of *R. damascena* Mill. ‘Kashan’ and ‘Hervy Azerbaijan’ through the activation of axillary buds. 

## 2. Materials and Methods

### 2.1. Plant Material

Experiments were carried out on ‘Kashan’ and ‘Hervy Azerbaijan’ Damask roses (*Rosa damascena* Mill.) in June 2021. The ‘Kashan’ genotype was prepared from two-year-old mother plants that were kept in the greenhouse of the North and Northwest Biotechnology Center of East Azerbaijan Province, Iran. On the other hand, 3-year-old mother plants of genotype ‘Hervy Azerbaijan’ were obtained from the greenhouse of the Technical and Professional Tissue Culture Center of East Azerbaijan province.

The ‘Kashan’ genotype shows a higher performance and more stability and adaptability than the ‘Hervy Azerbaijan’ genotype. ‘Kashan’ is a perennial plant with pale pink flowers and a growth period of at least 9 months per year. The approximate duration from germination to the end of flowering of this genotype is 75 days, and the average length of the flowering period is 25 days. The size of ‘Hervy Azerbaijan’ plants is smaller than that of the ‘Kashan’. The petal color of ‘Hervy Azerbaijan’ is dark pink and tends towards purple. The stem of ‘Hervy Azerbaijan’ (with approximately 80 cm height) is less thorny than that of ‘Kashan’ and its color is red in winter.

East Azerbaijan province is situated in the northwestern region of Iran, at an elevation of 1800 m above sea level, with coordinates of 46°25’ east longitude and 38°2’ north latitude, measured from the Greenwich meridian. Situated in a mountainous area, this province has a climate with mild summers and cold, long winters. 

The samples were taken in the form of 10–15 cm long semi-woody branches and, to maintain the moisture level and minimize potential damage, the specimens were carefully enveloped in a moist cloth and transported to the laboratory inside an insulated flask containing ice. After defoliation, the branches were cut into single-node explants of about 2 cm in length and used for in vitro culture.

### 2.2. Explants Disinfection

The explants were first washed with dishwashing liquid and Captan fungicide (3 g per 500 mL water for 20 min) prepared by the Exir-e-Keshavarzi Company, Yazd, Iran. In the subsequent step, the tissue samples underwent a one-hour immersion in running tap water. Following an initial wash, all samples underwent a one-minute treatment with 70% (*v*/*v*) ethyl alcohol, followed by a 5 min immersion in sodium hypochlorite (NaOCl) (trade mark of Active, Darou Pakhsh Co., Tehran, Iran) with a concentration of 20% (*v*/*v*). All disinfection procedures were carried out within a laminar flow hood cabinet. Ultimately, the samples underwent three washes in sterile distilled water, each lasting 5 min. 

### 2.3. Establishment of In Vitro Culture

**Figure 1:**
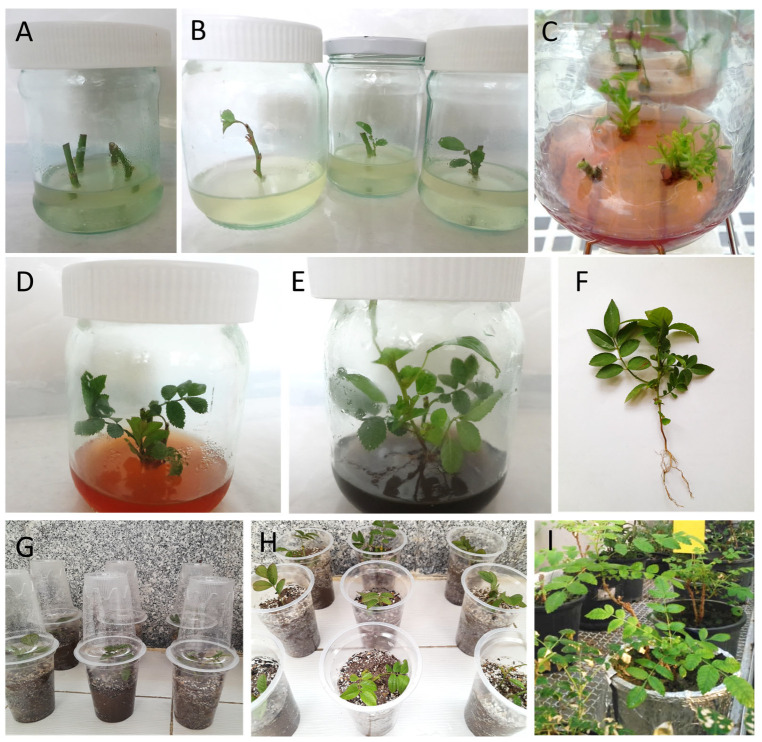
In vitro propagation stages (establishment, shoot proliferation, root induction, and acclimatization) of *Rosa damascenta* Mill. (**A**) Establishment stage, (**B**) growing explants in the establishment stage after 30 days of culture, (**C**) shoot proliferation stage in the multiplication medium augmented with 2 mg·L^−1^ BAP and 0.4 mg·L^−1^ GA_3_, (**D**) growing shoots after 60 days, (**E**) plantlets on the rooting medium after 30 days of culture, (**F**) rooted plantlet produced in ½ MS basal medium enriched with 2 mg·L^−1^ BAP together with 0.4 mg·L^−1^ GA_3_, (**G**,**H**) plantlets grown ex vitro in transparent plastic cups filled with autoclaved perlite and peat moss substrate (in a ratio of 2:1) in an acclimatization room, (**I**) acclimatized plantlets cultured in plastic pots filled out with soil, perlite and peat moss (in ratio of 2:1:1) in a greenhouse after 75 days. Scale bar: 10 mm.

Disinfected explants were placed inside sterile glass jars with a volume of 150 mL filled with 50 mL of basal MS [27] culture medium containing 3% (*w*/*v*) sucrose and 0.7% (*w*/*v*) agar (SIGMA Aldrich, Milwaukee, WI, USA) augmented with 1 mg·L^−1^ BAP, 0.2 mg·L^−1^ GA_3_, 0.1 mg·L^−1^ PG (SIGMA Aldrich, Milwaukee, WI, USA), 250 mg·L^−1^ Cefotaxime (Exir-e-Iran, Tehran, Iran), and 20 mg·L^−1^ ascorbic acid (Table 1, Figure 1A,B). The pH of the medium was adjusted to 5.6–5.8 before autoclaving at 105 kPa and 121 °C for 20 min. GA_3_ was cold disinfected with a sterilized filter. Vials containing 500 mg of cefotaxime powder were used to prepare the antibiotic solution. Sterile distilled water (2 mL) was injected into the vial with the powder using a sterile syringe. After dilution, 1 mL of cefotaxime (equivalent to 250 mg·L^−1^) was poured onto the sterilized culture medium. Also, filter-sterilized ascorbic acid was added to autoclaved culture media, to prevent phenolics secretion.

The cultures were grown at 24 ± 2 °C with a 16/8 h light/dark regime and 50–60 μmol·m^−2^·s^−1^ light intensity provided by cool-white fluorescent tubes. A subculture was performed after 15 days in similar vessels and culture conditions. After 30 days, adequate newly formed microshoots were used for the subsequent experiments. 

### 2.4. Shoot Proliferation and Root Induction

Following the establishment and initial growth of microshoots (3–4 cm long), nodal segments with axillary buds were used as secondary explants in the proliferation and rooting stages. MS medium containing 3% sucrose and 0.7% agar was used for shoot proliferation. The pH of the medium was adjusted to 5.6–5.8 as described above. The shoot proliferation media were supplemented with different concentrations of BAP (0, 0.5, 1, 1.5, 2 and 2.5 mg·L^−1^) and GA_3_ (0, 0.1, 0.2, 0.4, 0.8 and 1 mg·L^−1^). PG, antibiotic, and ascorbic acid were used in the same concentrations as in the establishment stage (Table 1). The cultures were kept in the same growth room as in the establishment stage for 30 days (Figure 1C,D). Half-strength (½ MS) basal medium with 3% sucrose and 0.7% agar was prepared for rooting (lasting 40 days). IBA at a concentration of 0.1 mg·L^−1^ and activated charcoal at a concentration of 20 mg·L^−1^ were added to the rooting media. BAP, GA_3_, PG, antibiotic, and ascorbic acid were used in the same concentrations as in the previous stages (Table 1, Figure 1E).

### 2.5. Acclimatization Process

The rooted microshoots (Figure 1F) were extracted from the culture jars, and any remnants of the culture medium adhering to the roots were removed by washing with lukewarm sterilized distilled water. Next, the bottom leaves of the young plants were carefully removed using scissors to facilitate their growth in pots. Plantlets were placed in a plastic cup filled with a mixture of autoclaved perlite and peat moss (in a 2:1 ratio) and watered with sterile water. A clear plastic cup was placed over the plantlets (Figure 1G,H). After 15 days, the plantlets were transferred to plastic pots (7.5 × 7.5 × 9 cm). One plantlet per pot was cultivated and pots were placed in an acclimatization room with 70 ± 5% relative humidity, 11/13 h light/dark regime (100 μmol m^−2^ s^−1^ light intensity), and 25 ± 2 °C temperature. After 20 days, the plantlets were transferred to the greenhouse (Figure 1I).

### 2.6. Experimental Design and Data Analysis

The factorial in vitro experiment was conducted in a completely randomized block design with 10 replications. Each experimental unit comprised five jars and three explants were cultured within each jar. During the microshoot proliferation step, bud induction time (bud break), number of green leaves, number of yellow leaves, shoot length, shoot number, shoot fresh weight, shoot dry weight, and regeneration coefficient (the number of lateral buds on the stem) were evaluated after 60 days of culture. The number of green and yellow leaves was recorded by manual counting. Rooting of microshoots was performed for 37 days, after which the time until root emergence, root number, root length (mean length of all regenerated roots per one microshoot), and the fresh and dry weight of roots were measured. To measure the dry weight of the shoots and roots, these organs were kept in an oven with a temperature of 200 °C for 20 min, immediately after the measurement of fresh weight. The greenhouse experiments were set in a completely randomized block design with three replicates and 72 pots with one plantlet (cultivar) in each. Data were subjected to the analysis of variance (ANOVA) and means were compared by Duncan’s test at *p* < 0.05 using the SAS ver. 9.1 software [28]. Data in graphs are presented as means ± standard deviations.

## 3. Results

After disinfection and establishment (with efficiency greater than 85%), the explants of both genotypes were transferred to the multiplication culture media. About 90% of explants regenerated shoots. 

**Figure 2:**
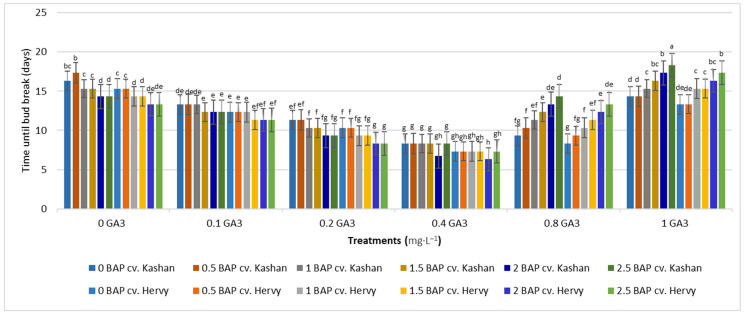
Mean comparison of the effect of different concentrations of GA_3_ and BAP on the time until bud break (days) of *Rosa damascena* Mill. genotypes ‘Kashan’ and ‘Hervy Azerbaijan’. Means marked with the same letter do not differ statistically according to Duncan’s test.

**Figure 3:**
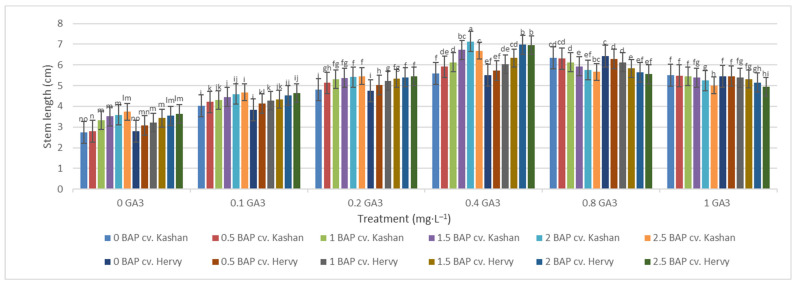
Mean comparison of the effect of different concentrations of GA_3_ and BAP on the stem length (cm) of *Rosa damascena* Mill. genotypes ‘Kashan’ and ‘Hervy Azerbaijan’. Means marked with the same letter do not differ statistically according to Duncan’s test. A comparison between both genotypes revealed that the highest number of shoots (4.29–4.78 per explant) was produced in the media supplemented with 2 mg·L^−1^ BAP and 0.4 mg·L^−1^ GA_3_ in ‘Hervy Azerbaijan’ and ‘Kashan’ genotypes, respectively (Figure 4). This number was approximately three-fold higher than the one obtained in the medium without PGRs. In general, ‘Hervy Azerbaijan’ produced less shoots than ‘Kashan’.

The shortest time until bud induction/bud break (6.33 days) was obtained in ‘Kashan’ explants cultured on the medium augmented with 2 mg·L^−1^ BAP with 0.4 mg·L^−1^ GA_3_ (Figure 2). In contrast, the longest time until bud break (18.33 days) was found in explants of ‘Hervy Azerbaijan’ cultured on the medium containing 2.5 mg·L^−1^ BAP with 1 mg·L^−1^ GA_3_ (Figure 2). Likewise, the longest stems (7.13 cm) were produced in the MS medium supplemented with 2 mg·L^−1^ BAP together with 0.4 mg·L^−1^ GA_3_ in the ‘Kashan’ genotype. The shoots of ‘Hervy Azerbaijan’ were usually shorter (maximal 6.96–6.97 cm in the medium with 2 or 2.5 mg·L^−1^ BAP and 0.4 mg·L^−1^ GA_3_), and the shortest stem length (2.7–2.8 cm) was obtained in the control medium for both genotypes (Figure 3).

**Figure 5:**
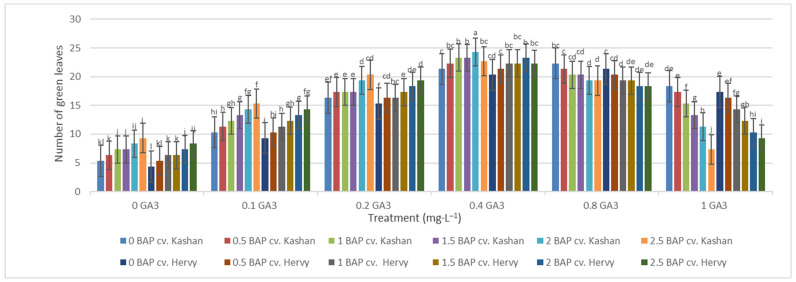
Mean comparison of the effect of different concentrations of GA_3_ and BAP on the number of green leaves (per explant) of *Rosa damascena* Mill. genotypes ‘Kashan’ and ‘Hervy Azerbaijan’. Means marked with the same letter do not differ statistically according to Duncan’s test.

**Figure 6:**
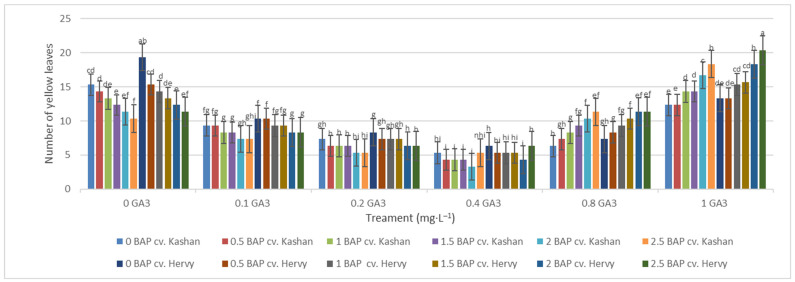
Mean comparison of the effect of different concentrations of GA_3_ and BAP on the number of yellow leaves (per explant) of *Rosa damascena* Mill. genotypes ‘Kashan’ and ‘Hervy Azerbaijan’. Means marked with the same letter do not differ statistically according to Duncan’s test.

The maximum number of green leaves (24.33 per explant) was counted in the treatment with 2 mg·L^−1^ BAP and 0.4 mg·L^−1^ GA_3_ in ‘Kashan’ (Figure 5). The number of green leaves was also high (23.66) in the same medium inoculated with ‘Hervy Azerbaijan’. On the other hand, the lowest number of green leaves (4.33–5.33 per explant) in both genotypes was counted in the culture medium without PGRs (Figure 5). The highest number of yellow leaves (18.33–20.33 per explant) was found in the medium containing 2.5 mg·L^−1^ BAP with 1 mg·L^−1^ GA_3_ in both genotypes studied. The number of yellow leaves was also high in the control medium with the ‘Kashan’ genotype (Figure 6). In general, the explants of both rose genotypes cultured on the media supplemented with 0.4 mg·L^−1^ GA_3_ (regardless of BAP concentration) had more green leaves and fewer yellow leaves. The opposite results were reported in plants grown in the control medium (Figure 5 and Figure 6).

**Figure 7:**
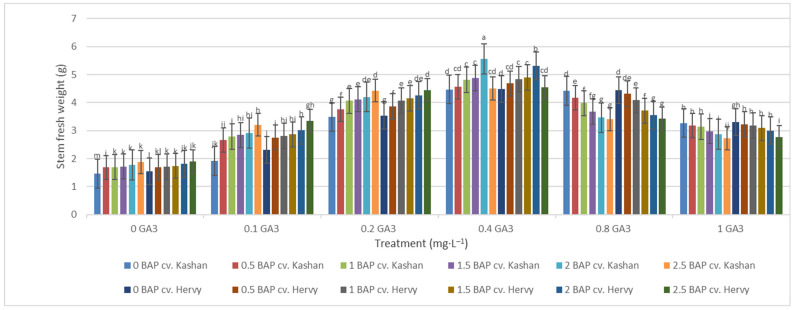
Mean comparison of the effect of different concentrations of GA_3_ and BAP on the stem fresh weight (g) of *Rosa damascena* Mill. genotypes ‘Kashan’ and ‘Hervy Azerbaijan’. Means marked with the same letter do not differ statistically according to Duncan’s test.

**Figure 8:**
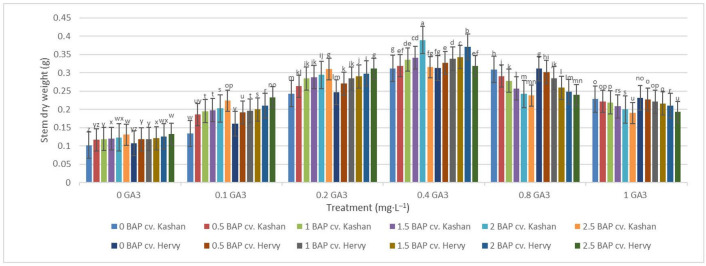
Mean comparison of the effect of different concentrations of GA_3_ and BAP on the stem dry weight (g) of *Rosa damascena* Mill. genotypes ‘Kashan’ and ‘Hervy Azerbaijan’. Means marked with the same letter do not differ statistically according to Duncan’s test.

The greatest fresh weight (5.31 and 5.56 g) of shoots was reported in the explants grown on the media supplemented with 2 mg·L^−1^ BAP with 0.4 mg·L^−1^ GA_3_ in ‘Hervy Azerbaijan’ and ‘Kashan’, respectively (Figure 7). The same media supplements were most effective in terms of plantlets’ dry weight (0.39 and 0.37 g per explant, respectively) (Figure 8). The lowest fresh (1.54 and 1.46 g) and dry weights (0.10 and 0.11 g) of the shoots were found in the plantlets grown on the medium without PGRs (control).

**Figure 9:**
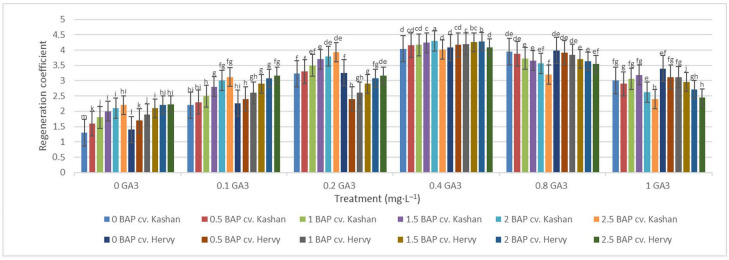
Mean comparison of the effect of different concentrations of GA_3_ and BAP on the regeneration coefficient of *Rosa damascena* Mill. genotypes ‘Kashan’ and ‘Hervy Azerbaijan’. Means marked with the same letter do not differ statistically according to Duncan’s test.

The highest regeneration coefficient was obtained in the medium augmented with 2 mg·L^−1^ BAP with 0.4 mg·L^−1^ GA_3_ in the ‘Kashan’ genotype (4.29) followed by ‘Hervy Azerbaijan’ (4.28). In general, the value of this parameter was high (over 4.0) in the media supplemented with 0.4 mg·L^−1^ GA_3_, regardless of BAP concentration. The lowest regeneration coefficient (1.30 and 1.40), on the other hand, was obtained in the control medium for both ‘Kashan’ and ‘Hervy Azerbaijan’, respectively (Figure 9). In general, explants cultured on the media without GA_3_ had a low regeneration coefficient (less than 2.23). 

**Figure 10:**
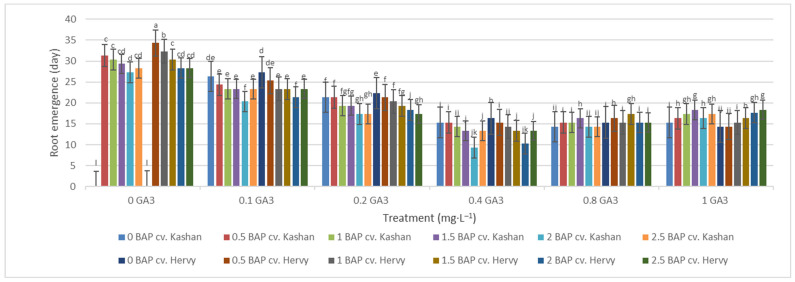
Mean comparison of the effect of different concentrations of GA_3_ and BAP on the time until root emergence (days) of *Rosa damascena* Mill. genotypes ‘Kashan’ and ‘Hervy Azerbaijan’. Means marked with the same letter do not differ statistically according to Duncan’s test.

The least time until root emergence (9.33 and 10.33 days) was observed in the medium augmented with 2 mg·L^−1^ BAP and 0.4 mg·L^−1^ GA_3_ in ‘Hervy Azerbaijan’ and ‘Kashan’, respectively (Figure 10). On the other hand, roots did not emerge for a very long time (even over a month) if GA_3_ was absent (or at low concentration) in the rooting medium. Roots were not produced in the control media. 

**Figure 11:**
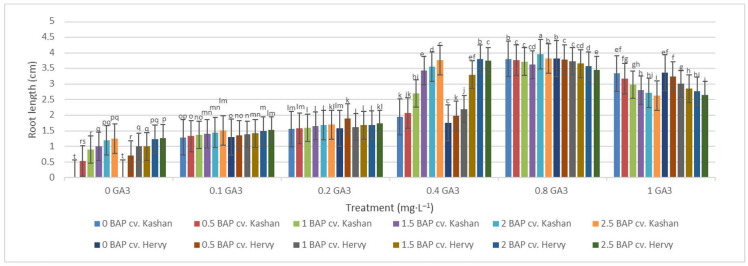
Mean comparison of the effect of different concentrations of GA_3_ and BAP on the root length (cm) of *Rosa damascena* Mill. genotypes ‘Kashan’ and ‘Hervy Azerbaijan’. Means marked with the same letter do not differ statistically according to Duncan’s test.

On the other hand, the longest roots were found in MS with 2 mg·L^−1^ BAP and 0.8 mg·L^−1^ GA_3_ in the ‘Kashan’ (3.96 cm per explant) and in the medium with 0.8 mg·L^−1^ GA_3_ only in the ‘Hervy Azerbaijan’ (3.82 cm). In general, plants produced on the media with low GA_3_ concentrations (0.0–0.2 mg·L^−1^) produced shorter roots (Figure 11).

**Figure 12:**
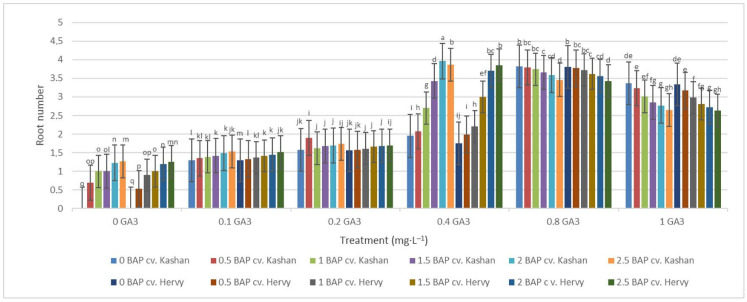
Mean comparison of the effect of different concentrations of GA_3_ and BAP on the root number of *Rosa damascena* Mill. genotypes ‘Kashan’ and ‘Hervy Azerbaijan’. Means marked with the same letter do not differ statistically according to Duncan’s test.

**Figure 13:**
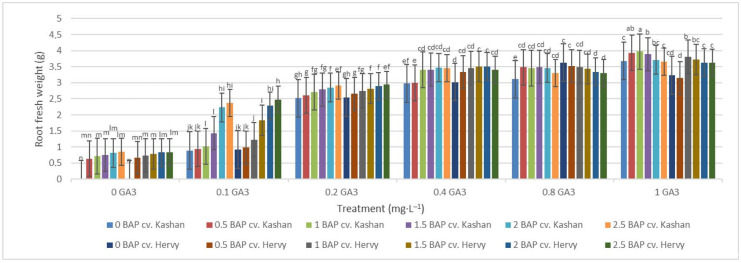
Mean comparison of the effect of different concentrations of GA_3_ and BAP on the root fresh weight (g) of *Rosa damascena* Mill. genotypes ‘Kashan’ and ‘Hervy Azerbaijan’. Means marked with the same letter do not differ statistically according to Duncan’s test.

**Figure 14:**
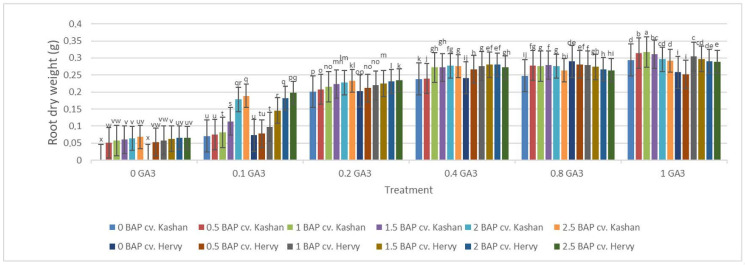
Mean comparison of the effect of different concentrations of GA_3_ and BAP on the root dry weight (g) of *Rosa damascena* Mill. genotypes ‘Kashan’ and ‘Hervy Azerbaijan’. Means marked with the same letter do not differ statistically according to Duncan’s test.

The highest root number (3.70–3.96 per explant) in both genotypes studied was found in the media enriched with 2 or 2.5 mg·L^−1^ BAP and 0.4 mg·L^−1^ GA_3_ or in the medium containing only 0.8 mg·L^−1^ GA_3_ (Figure 12). On the other hand, the highest fresh weight of ‘Kashan’ roots was produced in explants grown on the MS medium supplemented with 0.5 or 1 mg·L^−1^ BAP together with 1 mg·L^−1^ GA_3_. As for the ‘Hervy Azerbaijan’ genotype, the treatment with 1 mg·L^−1^ BAP and 1 mg·L^−1^ GA_3_ was most effective (3.81 g FW per explant) (Figure 13). The highest DW (0.31–0.32 g per explant) was found in the treatments with 1 mg·L^−1^ BAP together with 1 mg·L^−1^ GA_3_ in both genotypes (Figure 14).

Out of the 150 plants produced in the multiplication stage, 81 (54%) were successfully rooted (Figure 1F). Of these eighty-one samples, the adaptation of twenty-seven plants was related to the treatment of 2.5 mg·L^−1^ BAP without GA_3_, and five of the samples died in the adaptation stage, that is, 81.48% survived. The next twenty-seven plants were from the 2 mg·L^−1^ BAP treatment together with 0.2 mg·L^−1^ GA_3_, and three of these plants died in the adaptation phase, i.e., 88.88% survived. From the remaining 27 rose plants treated with 1 mg·L^−1^ BAP together with 0.8 mg·L^−1^ GA_3_, all samples (100%) survived. The plants produced in vitro exhibited developmental patterns similar to those of their in vivo origin mother counterparts. No phenotypical differences were observed.

## 4. Discussion

The present research reports a successful in vitro propagation system for *R. damascena* Mill. genotypes ‘Kashan’ and ‘Hervy Azerbaijan’. Previous studies revealed that PGRs play a vital role in enhancing the in vitro propagation efficiency of many species belonging to the Rosaceae family [7,29,30], which corresponds with our results. 

Among the cytokinins used, BAP has been found to be the most effective for axillary shoots multiplication in various *Rosa* genotypes [31,32]. For example, the highest multiplication rate (4.2–7.5 shoots per explant) in *R. canina* L was obtained using 1–2 mg·L^−1^ BAP [33,34]. A study on the in vitro propagation of *R. hybrida* L. demonstrated that the highest number of shoots, shoot length, and number of leaves were produced in the presence of 2 mg·L^−1^ BAP along with 0.1 mg·L^−1^ NAA [35]. The current study confirmed that BAP is effective for the proliferation of explants when used in moderately high concentrations (2 mg·L^−1^), especially in combination with GA_3_. 

Usually, the optimum concentration of PGRs for maximum shoot multiplication and root induction is not only species-specific, but also varies between genotypes, cultivars, and explant types, which is related to the different content of endogenous hormones [7,17]. Interestingly, in the present study, the highest number of shoots, in both genotypes studied, was produced in the same culture medium, i.e., MS containing 2 mg·L^−1^ BAP and 0.4 mg·L^−1^ GA_3_. This medium was also superior in terms of most of the other plant parameters we analyzed. The possibility of using the same medium composition for various genotypes highlights the utility of the micropropagation protocol developed here.

Our findings highlight the undeniable role of GA_3_ in improving all of the quantitative and qualitative traits of roses in vitro. GA_3_, particularly at the level of 0.4 mg·L^−1^, increased all of the positive characteristics and decreased all of the negative characteristics in both rose genotypes (‘Kashan’ and ‘Hervy Azerbaijan’). Gibberellins are known to promote the induction and elongation of microshoots [36,37] and to induce rhizogenesis [33]. The combination of GA_3_ and TDZ in the culture media of *Pterocarpus marsupium* Roxb. improved the germination percentage, multiplication, and subsequent elongation of shoots [38]. The use of 1 and 5 mg·L^−1^ GA_3_ resulted in 80.5 and 92.3% root induction in *Cynara scolymus* L. [36]. In the present study, GA_3_ stimulated rhizogenesis at even lower concentrations. Saks and Van Staden [39] reported that, when applied to the stems of cut carnations (*Dianthus caryophyllus* L.), GA_3_ delayed their senescence by reducing the climacteric peak of ethylene production. Likewise, Beevers [40] reported that gibberellic acid prevented yellowing and chlorophyll loss in nasturtium (*Tropaeolum majus* L.) leaf discs. This could explain why, in the present study, a lower number of yellow leaves and more green leaves were found in both genotypes of Damask rose in the optimal GA_3_ treatment. A comparison between our work and some other published papers, e.g., that by Nikbakht et al. [41] on the in vitro propagation of *R. damascene,* showed that the shoot multiplication rate obtained in our study (4.78 per explant) was higher. In *R. damascena* ‘Azaran’ and ‘Ghamsar’, BAP (1–2 mg·L^−1^), GA_3_ (0.1 mg·L^−1^), and NAA (0.1 mg·L^−1^) for ‘Azaran’ and the same concentrations of BAP and GA_3_ but without NAA for ‘Ghamsar’ provided the highest multiplication rates (about 2.5) and leaf parameters [41]. Shoot tips and axillary buds of *Rosa* ‘Konstancin’ (*R. rugosa* × *R. beggeriana*) developed into single shoots at a similar rate to ours (4.8 shoots per explant on the MS medium with 0.5 mg·L^−1^ BAP and 0.1 mg·L^−1^ GA_3_), but in a longer time period (2–4 weeks) [1].

The present study confirmed the beneficial effect of IBA on root induction and elongation in rose plants (‘Kashan’ and ‘Hervy Azerbaijan’). IBA is usually the most effective and frequently used auxin for rooting in woody plants, including Rosaceae [42,43,44,45]. However, root formation may be affected not only by the auxin type but also by its concentration [46]. Several studies showed that optimum root initiation and development were achieved when actively growing axillary buds were cultured on a medium supplemented with a high concentration of IBA [47,48]. The highest rooting percentage in three Iranian apricot (*Prunus armenica* L.) cultivars, ‘Ordubad’, ‘Shams’, and ‘Qaysi’, was achieved in a medium augmented with 4 mg·L^−1^ of IBA [29]. The maximum number of roots and root length in *Rosa hybrida* were obtained with 2 mg·L^−1^ IBA [35]. In peach (*Prunus persica* ‘Garnem’), 1.5 mg·L^−1^ IBA induced the maximum rooting rate (42.86%), maximum root number (6.33 per shoot), and longest roots (7.17 cm) [18]. As for *Physocarpus opulifolius* L., the best rooting was obtained at 1 mg·L^−1^ IBA in a half-strength MS medium [49]. In the present study, by using components such as PG and AC, we were able to achieve a high rooting efficiency of the Damask rose on the medium with a very low concentration of IBA (0.1 mg·L^−1^). 

Our study highlights the interaction between PG and other plant growth regulators in rose micropropagation. PG has been shown to increase shoot formation and somatic embryogenesis in various horticultural and grain crops [16]. Phloroglucinol, being a precursor in the lignin biosynthesis pathway, has the ability to control hyperhydricity through lignification. Moreover, its homologs act as auxin synergists or auxin protectors stimulating rooting [15]. This could explain why the simultaneous application of PG, BAP, and GA_3,_ so effectively stimulated the development of complete plants in the studied rose genotypes.

Morphological observations of both genotypes in the greenhouse conditions confirmed that propagated plants were true-to-type, which is highly beneficial. Explants cultured on artificial media are exposed to synthetic growth regulators and stress conditions that can stimulate genetic variations occurrence, particularly in the cells of callus. Genetic changes in the in vitro-produced plants have been a serious problem in the protocols based on indirect regeneration. Therefore, preserving the stability of plant material stands as one of the most vital objectives in commercial micropropagation [50,51]. Meristems, as well as shoot apical and lateral buds are relatively “safe” explants for the production of genetically stable plants [52]. The genetic stability of these explant types has been confirmed in several species, including roses [1,51,52,53]. The risk of producing off-type plants using other organs like roots, stems, and leaves is greater than in the case of meristems [45]. Therefore, in the present study, nodal segments with axillary buds have been used as primary and secondary explants. The micropropagation protocol developed in this study proved to be highly effective and practical, as no callus nor discernible morphological differences were observed between the in vitro-derived and mother plants.

## 5. Conclusions

Damask rose is primarily cultivated for industrial oil production, as its essential oil possesses notable pharmacological properties. Certain genotypes of Damask rose exhibit a higher potential for essential oil production, highlighting their significance in this regard. In this study, a rapid and efficient protocol was established for the in vitro propagation of two Damask rose genotypes, ‘Kashan’ and ‘Hervy Azerbaijan’. A combination of 2 mg·L^−1^ BAP with 0.4 mg·L^−1^ GA_3_ (in the presence of PG and IBA) enhanced the in vitro growth performance of both genotypes of *R. damascena* by improving most measured traits, particularly shoot and root number, the number of green leaves, and the regeneration coefficient. This protocol can be utilized in the large-scale production of oil-bearing roses.

## Figures and Tables

**Figure 4:**
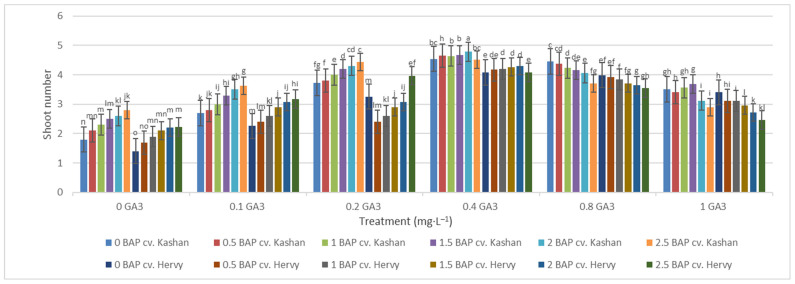
Mean comparison of the effect of different concentrations of GA_3_ and BAP on the shoot number of *Rosa damascena* Mill. genotypes ‘Kashan’ and ‘Hervy Azerbaijan’. Means marked with the same letter do not differ statistically according to Duncan’s test.

**Table 1:** Compounds added to the MS (for the establishment and shoot multiplication stages) and ½ MS (for rooting) basal medium for *Rosa damascena* Mill. ‘Kashan’ and ‘Hervy Azerbaijan’.

	Micropropagation Stages		
Additives (mg·L^−1^)	Establishment	Shoot Multiplication	Rooting
BAP	1	0, 0.5, 1, 1.5, 2 and 2.5	0, 0.5, 1, 1.5, 2 and 2.5
GA_3_	0.2	0, 0.1, 0.2, 0.4, 0.8 and 1	0, 0.1, 0.2, 0.4, 0.8 and 1
IBA	-	-	0.1
PG	0.1	0.1	0.1
Antibiotic	250	250	250
Ascorbic acid	20	20	20
Active charcoal	-	-	20
FeNaEDTA	-	100	100
Fe(OH)_3_	50	-	-

## Data Availability

The data are available by email at reasonable request.
